# Mission and Function of the Local Medical Journal

**Published:** 1905-03

**Authors:** 


### MISSION AND FUNCTION OF THE LOCAL MEDICAL JOURNAL.

Where there are a number of rival medical journals in the same State it is the duty of each physician to ascertain the best one, usually an easy task, and give that one his earnest support. In return for this support what does the medical journal owe to its subscribers? It owes them the prompt publication of all medical society reports and notices whenever such are furnished in proper season by the secretaries of these societies; and here let me say, in passing, that these societies should insist upon their secretaries sending reports of their meetings regularly to at least one of the journals published in the State. It owes them an active support in all matters pertaining to medical organization and medical legislation for the suppression of quackery and for the regulation of medical practice. It owes them a fair, honest and discriminating review of all new medical books, by men competent to write such reviews, so that its readers may know which of the new books it will pay them to buy, and which ones to avoid buying. A most valuable feature of the local medical journal and one which can not fail to be of great service to its subscribers if the work is well done, is the abstracting of the important contributions to the different departments of medicine and surgery, taken from current journals which its readers are, for the most part, not likely to have access to.

The local medical journal has a very important part to play in the uniting and organizing and holding together the members of the medical profession in its own territory, in voicing their sentiments on all matters of general interest to the profession, and also in bringing the medical men of different parts of the country into closer touch with each other. Many an important contribution to medical literature has been made in a paper read before some little unknown local medical society, which would perhaps never have been printed had not the editor of the local medical journal pursued its author until he was finally induced to put his manuscript into shape for printing and send it in for publication. Many rare and important observations are recorded in the local journals, which, were it not for the existence of these medical journals, would never be recorded. Such papers and clinical reports frequently give rise to correspondence between medical men in different parts of the country and form the starting-point of pleasant and valuable friendships between physicians who might otherwise have never known each other.

The local medical journal is a perfectly legitimate and a convenient medium by means of which those who are engaged in certain special lines of practice may make themselves and their work known to the profession of the State, either by means of papers and case reports, or by means of a standing card in the advertising pages of the journal. This latter method has never found favor in this part of the country, although in many places it is a common practice and there can be no ethical or other objection to it.—*Burnside Foster, Editor St. Paul Medical Journal.*

